# Histological Evaluation of Polyacid-Modified Composite Resin and Conventional Composite Resin Used for Primary Molars Restoration

**DOI:** 10.3390/dj12110343

**Published:** 2024-10-28

**Authors:** Omar A. El Meligy, Hisham I. Othman, Shahad N. Abudawood, Amani A. Al Tuwirqi, Madawi Faisal Alkeheli, Tarek R. Abdelrehim, Dalia M. Talaat

**Affiliations:** 1 Pediatric Dentistry Department, Faculty of Dentistry, King Abdulaziz University, Jeddah 21589, Saudi Arabia; sabudawood@kau.edu.sa (S.N.A.); aaltuwirqi@kau.edu.sa (A.A.A.T.); 2 Pediatric Dentistry and Dental Public Health Department, Faculty of Dentistry, Alexandria University, Alexandria 21131, Egypt; dalia.talaat@dent.alex.edu.eg; 3 Oral Diagnostic Sciences Department, Faculty of Dentistry, King Abdulaziz University, Jeddah 21589, Saudi Arabia; hothman@kau.edu.sa (H.I.O.); malkahele@kau.edu.sa (M.F.A.); 4 Oral and Maxillofacial Prosthodontics Department, Faculty of Dentistry, King Abdulaziz University, Jeddah 21589, Saudi Arabia; tabdalrahem@kau.edu.sa

**Keywords:** compomer, composite resin, primary molars, restoration

## Abstract

**Background:** The present study evaluated the histological outcomes of two dental restorative materials, polyacid-modified composite resin (compomer) and conventional composite resin, in the primary molars of puppies. **Materials and Methods:** Twenty sound primary molars in four puppies were used. The puppies were rendered unconscious using general anesthesia. Similar cylindrical Class V cavities were prepared in 16 of the 20 selected primary molars. The teeth were divided into three groups: Group I: Eight cavities were restored with compomer; Group II: Eight cavities were restored with conventional composite resin; Group III: Four teeth remained untreated and were used as controls. In Groups I and II, four teeth were examined histologically after 2 weeks and the other four after 6 weeks. The histological findings were analyzed and compared to determine the effects of each type of resin material on the dentine and the pulp. **Results:** At 6 weeks, the specimens tested for compomer showed obvious destructive changes in the central region and the region of the pulp adjacent to the cavity. The specimens tested for conventional composite resin revealed, at 6 weeks, massive destruction of the pulp tissues and abscess formation was observed. All the specimens tested in the control group showed normal cellularity, normal vascularity, and proper alignment of odontoblast cells. **Conclusions:** The teeth restored with compomer demonstrated more favorable pulpal reactions when compared with the teeth restored with conventional composite resin after 6 weeks.

## 1. Introduction

Dental restorative materials play a vital role in pediatric dentistry, particularly in primary tooth restorations where the material’s biocompatibility and properties are crucial. Among the commonly used options are compomers and composite resins. Compomers, a hybrid of composite and glass ionomer materials, are valued for their fluoride release and favorable biocompatibility. In contrast, composite resins, known for their strength and aesthetic appeal, are primarily composed of methacrylate-based substances like Bis–GMA and TEGDMA. While composite resins have been widely used due to their durability and appearance, concerns about their biocompatibility, specifically their potential cytotoxicity in pulpal tissues, have emerged in recent years [1].

Evaluating the histological properties of composite resins and compomers is crucial to assessing their biocompatibility and how they interact with surrounding tissues. These materials, frequently used in dental restorations, can elicit varying cellular responses in pulp, connective tissues, and bone. Composite resins are favored for their aesthetic appeal and adhesive strength, but their biocompatibility remains a subject of concern. It has been found that when the composite resin Dualcim was implanted in rats, it caused moderate inflammation, which subsided over time, suggesting the material’s acceptable biocompatibility for dental use [2]. Similarly, in a comparison of different restorative materials through immunohistochemical and ELISA analyses, it has been found that composite resins triggered higher inflammatory responses in fibroblast cells than compomers and glass ionomer cements, which were better tolerated by tissues [3].

Compomers, as hybrid materials that merge the properties of composites and glass–ionomer cements, have demonstrated superior biocompatibility in various studies when compared with traditional composite resins. In a study comparing the histological responses of connective tissues with compomers and composite root-end fillings, it was found that compomers induced a lower inflammatory response and promoted better tissue healing than composites [4]. Similarly, compomers, when compared with silver amalgam, caused fewer adverse reactions, and a histological analysis revealed better tissue integration [5].

Further supporting this was another study, exploring the effects of compomers, resin-modified glass–ionomer cements, and composites on periodontal tissues in dogs. The findings showed that compomers resulted in reduced fibroblast adhesion and inflammation, making them more suitable for periodontal applications [6]. Additionally, the advantages of compomers over conventional glass–ionomer cements in caries inhibition were emphasized. The study demonstrated that compomers offered better marginal integrity and biocompatibility, particularly for primary teeth [7].

The future of compomers in dentistry is promising due to their ease of manipulation and versatility in clinical practice. Recent advancements in dental materials are concentrating on enhancing both the mechanical and aesthetic properties of these materials while ensuring that they remain easy to use for dental practitioners. Compomers are particularly favored for their fluoride-release capabilities and user-friendliness, making them especially popular in pediatric dentistry where quick, efficient, and reliable materials are essential [8]. Current development trends point toward future improvements that will likely enhance compomers’ handling properties and clinical performance. These enhancements are expected to make compomers an even more reliable option for long-term dental restorations in primary teeth [9].

While previous studies have evaluated the biocompatibility of restorative materials, such as conventional composite resin and compomers, in human dental applications, limited research has explored their biological impact in developing dentition under experimental conditions [10]. Animal studies allow for a controlled histological analysis that can reveal tissue responses and the potential long-term effects in a developing dentition, which cannot be ethically or practically studied in human patients.

The aim of the present research was to study the effect of polyacid-modified composite resin and conventional composite resin on the dentine and pulp of primary molars in puppies. The null hypothesis tested was that there would be no difference in the effects of these two materials on the dentine and pulp tissues.

## 2. Materials and Methods

### 2.1. Ethical Approval

The Faculty of Dentistry, Alexandria University Ethical Committee approved the research protocol (IRB NO:00010556–IORG 0008839), and the Manuscript Ethics Committee number was 0982-09/2024. This study was conducted in compliance with all the policies of appropriate animal care at Alexandria University.

### 2.2. Procedure

Twenty sound primary molars in four puppies were used. The puppies were 3–6 months old, as this is the age when primary molars are most similar to children’s teeth and can undergo a histological evaluation for pulpal responses. The puppies were generally anesthetized with an intramuscular injection of 5% ketamine (10 mg/kg body weight, Veterinary Ketalar 50) and with an intravenous injection of 5% sodium pentobarbital (20 mg/kg body weight). The surface of each tooth was scaled and disinfected with 30% hydrogen peroxide solution [11]. Cylindrical Class V cavities were prepared on 16 primary molars. The cavosurface margins were kept in enamel. The diameter of each cavity was 1.8 mm corresponding to the size of the bur, while the depth was 0.5 mm [12]. The depth was kept standard by means of a hard rubber tube insert, smaller in diameter to the bur. This covered all the working length of the bur except the lower 0.5 mm. The burs were mounted on a high-speed handpiece (KaVo MASTERtorque LUX M8900L, KaVo Dental GmbH, Biberach, Germany) and were changed periodically every four cavities. One primary molar in each puppy remained untreated and was used as a control [13].

For the histological examination, the teeth were divided into three groups:

Group I: Eight cavities were filled with the polyacid-modified composite resin “Dyract” (Dentsply, DeTrey, Konstanz, Germany).

Group II: Eight cavities were filled with the conventional composite resin “Degufill H” (Degussa, Frankfurt, Germany).

Group III: Four teeth remained untreated and were used as controls.

### 2.3. Tooth Filling

The manufacturer’s instructions concerning the manipulation of the two materials were strictly followed. The cavities were cleaned with a water spray, and excess moisture was removed using oil-free compressed air. For the “Dyract” restorations, a layer of “Dyract–PSA Prime/Adhesive” was applied to the exposed dentine and enamel surfaces and left undisturbed for 30 s. Any excess solvent was removed with an air syringe, and the layer was light-cured for 10 s. To ensure full coverage, a second layer of “Dyract–PSA Prime/Adhesive” was applied using the same method as the first. “Dyract” was then placed into the cavity with the manufacturer’s recommended dispensing instrument, in increments of 3 mm or less, with each increment light-cured for 40 s to reduce polymerization shrinkage.

For the “Degufill H” restorations, the entire cavity was treated with Degufill Etchant (37% orthophosphoric acid) for 60 s, rinsed for 30 s, and dried with oil-free compressed air. “Degufill Bond” was then applied to all the cavity surfaces with a brush, spread with an oil-free air spray, and light-cured for 20 s. “Degufill H” was dispensed directly into the cavity with a syringe and light-cured for at least 40 s. The final finishing was performed using a carbide finishing bur [14].

Postoperatively, the puppies were housed in a controlled environment where they received adequate veterinary care. This included regular check-ups to ensure that their overall health was optimal, and that no other factors (like infections or diseases) could affect the study’s outcomes. They were put on a balanced diet, which consisted of meat, milk, and bread with broth until the time of sacrifice.

### 2.4. Histological Evaluation

In both Groups I and II, four teeth were examined after 2 weeks and the other four after 6 weeks. This served as an observation of the short- and long-term effects of each type of the two resin materials on the dentine and pulp. The animals were sacrificed after a postoperative period of 2 and 6 weeks, respectively. They were euthanized in a humane manner to minimize pain and distress. A common method involved administering an overdose of a general anesthetic, such as pentobarbital, which ensures a quick and painless procedure. The teeth, along with the surrounding bone and tissue, were removed as a block using a water-cooled diamond disc (Dentsply Sirona, York, PA, USA) and fixed in neutral buffered formalin for preparation for light microscopic examination under a light microscope (Olympus Corporation, Tokyo, Japan) at 100× magnification.

### 2.5. Specimen Preparation

The specimens were decalcified in 5% trichloroacetic acid. Thereafter, they were washed, dehydrated in ascending grades of alcohol 50%, 70%, 90%, and 100%, then put in xylin and embedded in paraffin. Serial sections at 5 µm were cut on a rotatory microtom (Leica Microsystems, Wetzlar, Germany) through the cavities on the longitudinal axis of the teeth and stained with hematoxylin and eosin.

The histological findings were analyzed and compared to determine the effects of each type of the resin material on the dentine and pulp.

## 3. Results

### 3.1. Group I (Polyacid-Modified Composite Resin)

**Figure 1:**
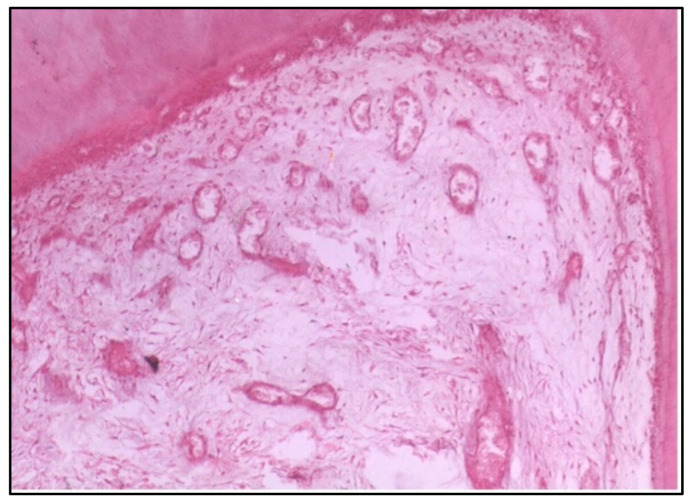
Longitudinal section (LS) of the pulp (Group I, 2 weeks), showing disorganization of odontoblasts and an increase in the vascular supply to the pulp with the budding of new capillaries (H & E Stain, ×100).

At 2 weeks, the specimens exhibited notable changes in the pulp tissue. The odontoblasts, which are the cells responsible for forming dentine, showed disorganization, indicating a disruption in their usual structure and function. There was also an increase in the vascular supply, suggesting an inflammatory response. The budding of new capillaries likely reflects the body’s attempt to repair or regenerate tissue. This reaction is typical of a mild to moderate inflammatory process following irritation or injury caused by the restorative material (Figure 1).

**Figure 2:**
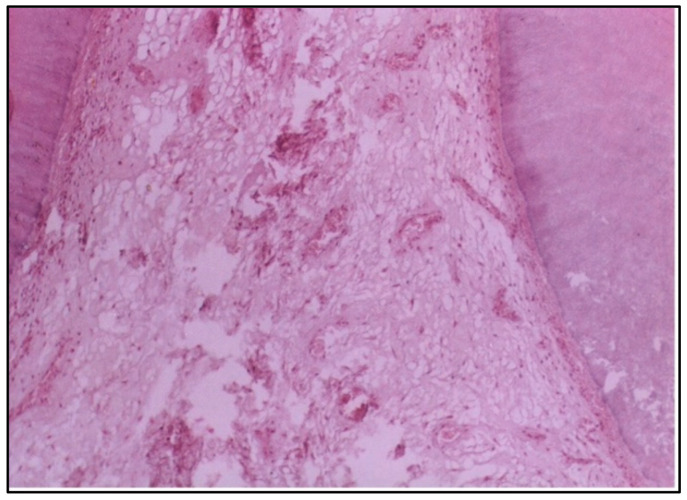
LS of the pulp (Group I, 6 weeks) showing obvious destructive changes in the central region and the side of the pulp adjacent to the cavity (H & E Stain, ×100).

At 6 weeks, the pulp tissue exhibited more pronounced destructive changes. The central region of the pulp and the area adjacent to the cavity showed degeneration, indicating that the pulp tissue was unable to recover and was undergoing significant damage (Figure 2).

### 3.2. Group II (Conventional Composite Resin)

**Figure 3:**
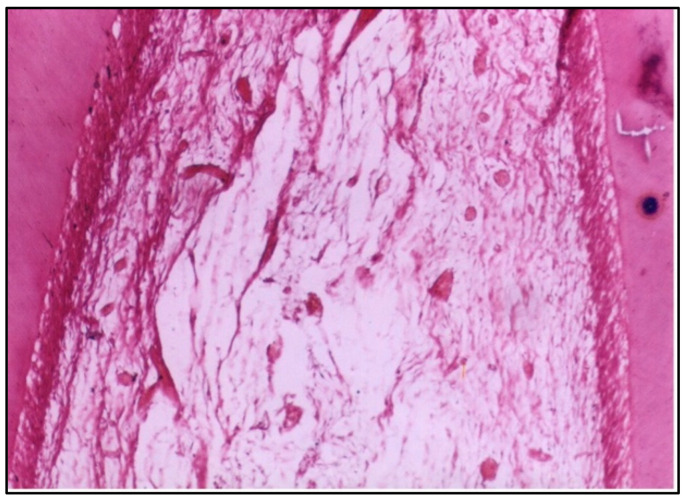
LS of the pulp (Group II, 2 weeks) showing moderate disorganization of the pulp tissue. Note that the cells and fibers are condensed at one side and loosely arranged in the center (H & E Stain, ×100).

**Figure 4:**
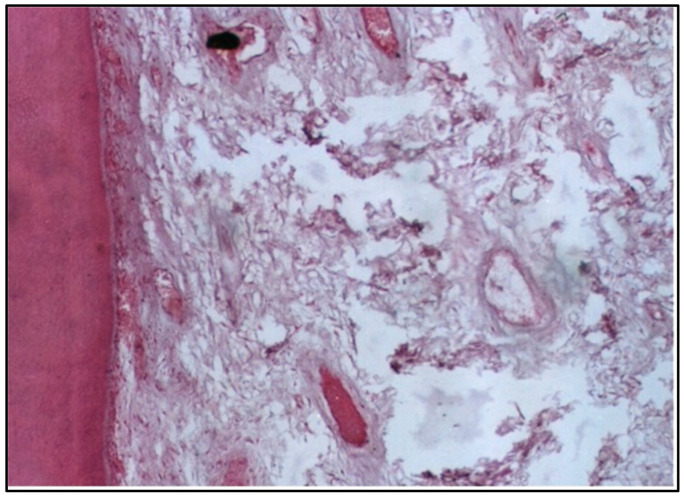
LS of the pulp (Group II, 2 weeks) showing a generalized appearance of disorganization with microabscess formation (H & E Stain, ×100).

At 2 weeks, the pulp tissue displayed moderate disorganization, but the pattern of damage was more localized compared with Group I. The cells and fibers at one side of the tissue were condensed, which could indicate fibrosis, a common reaction to injury. Meanwhile, the central area was more loosely arranged, reflecting ongoing inflammation (Figure 3). Notably, one of the specimens had a microabscess formation, signifying localized pus accumulation due to infection or significant inflammation, which was more severe than the vascular changes observed in Group I (Figure 4).

**Figure 5:**
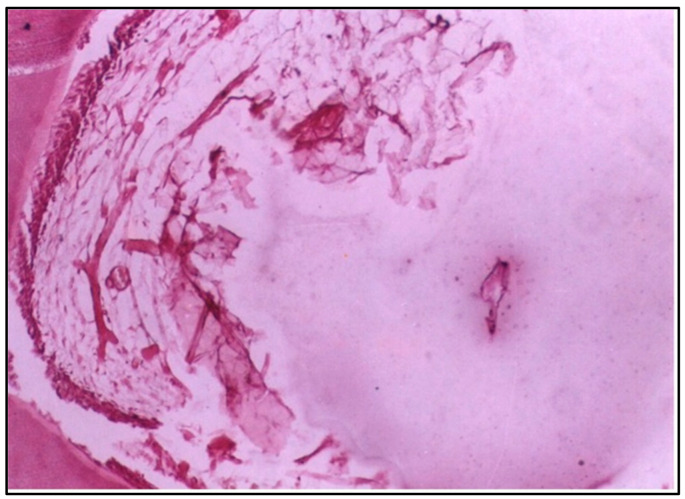
LS of the pulp (Group II, 6 weeks) showing massive destruction of the pulp tissue with an abscess formation (H & E Stain, ×100).

At 6 weeks, the damage was even more severe, with massive destruction of the pulp tissue, including the formation of a large abscess. This level of destruction indicates that the pulp could not withstand the long-term effects of the conventional composite resin, leading to extensive tissue necrosis and infection (Figure 5).

### 3.3. Group III (Control Group)

**Figure 6:**
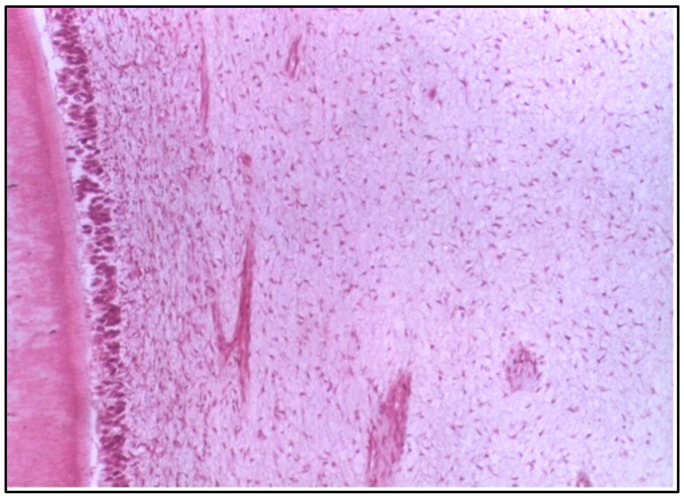
LS of the pulp in situ (control group) showing normal cellularity, normal vascularity, and proper alignment of the odontoblast cells (H & E Stain, ×100).

At 2 and 6 weeks, the specimens in the control group, which were not exposed to any restorative material, showed normal tissue architecture throughout the observation periods. The odontoblast cells were properly aligned, and the vascularity and cellularity of the pulp were normal. This confirms that the experimental changes in Groups I and II were due to the materials used and not due to natural tissue variation or a response to the experimental conditions (Figure 6).

## 4. Discussion

The null hypothesis was rejected, since the teeth restored with compomer demonstrated more favorable pulpal reactions when compared with the teeth restored with conventional composite resin.

It has been generally accepted that many of the materials used in restorative dentistry may harm the dental pulp. There are good reasons for expecting the polyacid-modified composite resin to be less irritating towards dental pulp. The histological evaluation of the dental materials, polyacid-modified composite resin (compomer), and conventional composite resin provides critical insights into their biocompatibility and the tissue responses they elicit.

The present study investigated the effects of these materials on the dentine and pulp tissue in the primary molars of puppies over time, with significant findings observed at 2 and 6 weeks. Puppies, particularly those at a specific stage of dental development, were chosen because their primary teeth bear a close resemblance to human primary molars in structure and function. This allowed researchers to simulate the effects of restorative materials in an animal model that closely mimics human pediatric dentistry. Medium-sized breeds were chosen for the present study because their teeth are similar in size and composition to human primary molars, providing reliable comparative data [15].

Cylindrical Class V cavities were prepared on 16 primary molars of puppies. The cavosurface margins were kept in enamel. It is important to consider that Class V restorations in clinical practice are often placed with margins on cement rather than enamel or dentin, which can influence adhesion and the overall quality of the restoration. A recent review highlighted the implications of using bulk-fill resin-based composites in sealing cavities with margins in radicular cementum [16]. The analysis emphasized the need for the careful consideration of restorative materials and the conditions under which they are applied, as they may significantly affect the outcomes related to adhesion and potential pulp health. Given these insights, the destructive changes observed in the pulp tissue following the use of compomer and conventional composite resin may be exacerbated by the differences in how these materials adhere to the tooth structure when the margins are located on cement. This underscores the importance of understanding the material properties and the clinical context of restoration placement when evaluating histological outcomes.

Regarding polyacid-modified composite resin, at 2 weeks, the specimens tested exhibited disorganization of odontoblasts and increased the vascular supply, indicated by the budding of new capillaries. This suggests an initial inflammatory response, possibly due to the material’s composition or the polymerization process. The increased vascularity can be interpreted as a reparative attempt by the pulp tissue to maintain homeostasis and facilitate healing [17].

At 6 weeks, more severe changes were evident, with destructive alterations in the central pulp region and the areas adjacent to the cavity. These findings could indicate a failure to adapt to the material or prolonged inflammatory response, potentially due to the prolonged exposure to residual monomers or other components of the resin [18]. In addition, the destructive changes in the pulp adjacent to the cavity at 6 weeks may be attributed to the proximity of the restorative material to the pulp. It is conceivable to suggest that the cavities used in the present study, although of a depth of 0.5 mm, are considered deep because of the small thickness of the enamel and dentine of puppies’ primary teeth, which is about 0.7 mm.

Concerning the conventional composite resin, the specimens showed moderate disorganization of the pulp tissue at 2 weeks, with one specimen presenting microabscess formation, indicating localized areas of necrosis and acute inflammation. This aligns with previous studies highlighting the cytotoxic potential of certain composite resins, often attributed to their chemical constituents, such as Bis–GMA and TEGDMA [10].

At 6 weeks, the situation had worsened, with massive pulp tissue destruction and abscess formation observed. This suggests an inability of the pulp tissue to recover from the initial insult, leading to progressive necrosis and infection. The persistence of such reactions over time might be related to factors such as incomplete polymerization and the leaching of unreacted monomers [19].

In the present study, three possibilities were the cause of pulpal irritation. The first one might be trauma to the pulp during cavity preparation. This may have been responsible for the moderate pulpal response in the 2-week period. The second cause might be the bacterial contamination during the filling procedure, which may also account for the bacteria found in the short observation period and the severe inflammatory response revealed in the longer observation period. The third possibility might be from microleakage.

The severe pulp response observed in one specimen with conventional composite resin was manifested by the presence of dense inflammatory cell accumulation and localized abscess formation beneath the cavity. This observation could be due to the presence of bacterial contamination during the filling procedure and/or microleakage and the long–standing bacteria and their endotoxins at the restoration/tooth interface. This finding has also been observed by Riberio et al. [20]. In contrast, the control group exhibited normal cellularity, vascularity, and odontoblast alignment at both 2 and 6 weeks. This highlights the natural regenerative capacity of the pulp tissue in the absence of irritants, reaffirming the importance of selecting biocompatible materials for restorative procedures [21].

The observed differences in tissue responses between polyacid-modified composite resin (compomer) and conventional composite resin may be attributed to several key factors related to their chemical composition and behavior during polymerization. Firstly, chemical composition plays a significant role in the tissue responses. Compomer contains acidic groups that can interact with dentin, potentially leading to less aggressive chemical reactions compared with conventional composite resin, which releases more reactive monomers such as bisphenol A–glycidyl methacrylate (Bis–GMA) and triethylene glycol dimethacrylate (TEGDMA). These monomers can cause more irritation to the pulp tissue, contributing to the observed pulp destruction in conventional composite restorations [10]. Secondly, polymerization shrinkage is a critical factor. Conventional composite resin tends to undergo more significant polymerization shrinkage, creating stress at the cavity margins, which may lead to microleakage and bacterial infiltration. This process can contribute to the observed abscess formation and pulp tissue damage. In contrast, compomer materials generally exhibit lower shrinkage rates, which might explain the more favorable pulpal reactions [22]. Lastly, the release of monomers during polymerization can differ between the two materials. Conventional composite resins are known to release residual monomers that can diffuse through the dentin and irritate the pulp. The compomer material, with its unique ion-releasing properties, may release fewer harmful monomers, potentially reducing pulpal inflammation [23].

The degree of polymerization is crucial, as insufficient polymerization can result in residual unreacted monomers, which may lead to increased cytotoxicity and inflammation in the pulp tissue. Prolonged polymerization time and optimal light intensity can enhance the conversion of monomers to polymers, reducing the likelihood of adverse pulp responses. In the present study, we ensured that both materials were subjected to the manufacturer’s recommended polymerization protocols [24]. Additionally, the use of adhesives can significantly impact the dental permeability. Adhesives that penetrate dentin create a hybrid layer, which can either enhance or reduce the permeability of the dentin to external substances. Increased permeability may facilitate the diffusion of unreacted monomers or bacteria into the pulp chamber, potentially exacerbating inflammatory responses and tissue damage. In our findings, the interplay between the adhesive properties and the restorative materials may have contributed to the observed histological changes, underscoring the importance of selecting appropriate adhesive systems in dental restorations [25].

The difference in the results between the two resin materials used in the current study may be attributed to etching of the vital dentine, prior to the placement of the composite resin. Etching the vital dentine may result in increased pulp irritation by facilitating the penetration of irritants into the tubules. This explanation is consistent with the findings of other investigations [26,27].

This study provides valuable insights into the histological outcomes of the two resin materials; however, several limitations must be considered. The use of a puppy model may not accurately reflect human dental conditions, as the differences in enamel and dentin thickness, pulp anatomy, and dental maturity can limit the applicability of the findings. Additionally, the small sample size of 20 primary molars may affect the reliability and statistical power of the results, necessitating a larger sample for more generalizable conclusions. The 6-week follow-up is relatively short, leaving the long-term effects of the restorative materials on pulpal health and tissue responses unknown, which highlights the need for longer evaluations to assess durability and potential late complications. Caution should be taken when extrapolating the findings to human dentistry, as differences in anatomical and biological responses may exist, necessitating further research in human studies for validation. Finally, the study may be influenced by biases related to design and the inherent variability among animal subjects, and future studies should aim to control for these factors through randomization and blinding to enhance reliability. A key limitation of the present study is the absence of a formal power analysis, which restricts the ability to generalize the findings and may reduce the sensitivity to detect subtle differences between the experimental groups.

## 5. Conclusions

The present findings indicate that conventional composite resin resulted in significant pulpal necrosis and abscess formation, demonstrating a more detrimental effect on pulp tissue. In contrast, the polyacid-modified composite resin (compomer) produced a milder initial inflammatory response.

The results suggest that compomer may be a less irritating alternative for the restorations of primary teeth based on short-term data; however, additional studies are necessary to confirm its long-term safety and efficacy.

## Data Availability

Data are contained within the article.
